# Chromium (III) removal by perennial emerging macrophytes in floating treatment wetlands

**DOI:** 10.1038/s41598-023-49952-y

**Published:** 2023-12-16

**Authors:** Nicole Nawrot, Ewa Wojciechowska, Muhammad Mohsin, Suvi Kuittinen, Ari Pappinen, Karolina Matej-Łukowicz, Katarzyna Szczepańska, Agnieszka Cichowska, Muhammad Atif Irshad, Filip M. G. Tack

**Affiliations:** 1 Faculty of Civil and Environmental Engineering, Gdansk University of Technology, Narutowicza 11/12, 80-233 Gdansk, Poland; 2 School of Forest Sciences, University of Eastern Finland, Yliopistokatu 7, P.O. Box 111, 80100 Joensuu, Finland; 3 Department of Environmental Protection, Gdynia Maritime University Maritime Institute, Gdynia, Poland; 4 Department of Environmental Sciences and Engineering, Government College University, Faisalabad, 38000 Pakistan; 5 Department of Environmental Sciences, The University of Lahore, Lahore, 54590 Pakistan; 6 Department of Green Chemistry and Technology, Faculty of Bioscience Engineering, Ghent University, 9050 Ghent, Belgium

**Keywords:** Environmental sciences, Environmental chemistry, Environmental impact

## Abstract

Floating treatment wetlands (FTWs) are a sustainable solution to treat polluted water, but their role in chromium (Cr(III)) removal under neutral pH conditions remains poorly understood. This study evaluated the potential of FTWs planted with two perennial emergent macrophytes, *Phragmites australis* and *Iris pseudacorus,* to remove Cr(III) and nutrients (N and PO_4_-P) from water containing 7.5 mg/L TN, 1.8 mg/L PO_4_-P, and Cr(III) (500, 1000, and 2000 µg/L). Within 1 h of exposure, up to 96–99% of Cr was removed from the solution, indicating rapid precipitation. After 50 days, Phragmites bound 9–19% of added Cr, while Iris bound 5–22%. Both species accumulated Cr primarily in the roots (BCF > 1). Biomass production and growth development were inhibited in Cr treatments, but microscopic examination of plant roots revealed no histological changes at 500 and 1000 µg/L Cr, suggesting high resistance of the tested species. At 2000 µg/L Cr, both species exhibited disruptions in the arrangement of vessel elements in the stele and increased aerenchyma spaces in *Phragmites*. At the end of the experiment, 70–86% of TN and 54–90% of PO_4_-P were removed.

## Introduction

Chromium (Cr) is a naturally occurring element that is also released into the environment through human activities such as industrial processes (e.g., leather tanning), fossil fuel combustion, and the production of steel, glassware, second-generation fertilizers, among others^1^. The abundance of Cr in the Earth’s crust ranges from 5 to 3400 mg/kg, with higher concentrations in igneous rocks^2^. Chromium can be easily transferred from soil and air to surface and groundwater^3^, posing a risk to aquatic organisms and humans.

Natural baseline Cr concentrations in surface water range from 0.5 to 2 µg/L for total Cr and between 0.02 and 0.3 µg/L for dissolved Cr^4^. Rainwater typically contains 0.2 to 1 µg/L of Cr^5^. The most precautionary European countries (Norway and Poland) have a maximum allowable Cr concentration in drinking water of 50 µg/L^6,7^.

In 2021, the Environmental Protection Agency of Poland reported Cr(VI) concentrations ranging from 0 to 7800 µg/L in some surface waters of lakes and rivers in Poland^8^. Wastewater discharge limits of Cr vary by country. The maximum discharge limits for Cr(VI) are 1000 µg/L in Belgium and 2000 µg/L in the Netherlands, while Spain has a limit of 5000 µg/L for total Cr. The environmental policies of Greece, Austria, and Denmark only regulate total Cr concentrations in wastewater, whereas the maximum allowable limit for Cr(VI) in Poland, Germany, and Hungary is 500 µg/L^7^.

The most common oxidation states of Cr in the terrestrial environment are + III and + VI. Trivalent Cr(III) is an essential element for animals and humans, but hexavalent Cr(VI) is carcinogenic and mutagenic, with several interconnected negative effects on the human circulatory, respiratory, and digestive systems^1^. In natural environments, Cr(VI) can be easily reduced to Cr(III) by organic matter, Fe(II), and sulphides. However, Cr(III) can also be oxidation back to Cr(VI) by naturally occur various manganese oxides^9,10^. Therefore, effective and sustainable methods of chromium removal from water, wastewater and surface runoff should be developed.

Many traditional approaches to removing Cr from water and wastewater rely on physico-chemical methods (chemical reduction, adsorption, electrocoagulation). These methods can be expensive to implement and operate, and they can also produce secondary pollution^11^. Bioremediation offers a viable alternative with a lower environmental footprint and lower costs. Engineered bioremediation systems, such as constructed wetland and floating treatment wetlands, have received a lot of attention in recent years^12^.

Floating treatment wetlands (FTWs) are surface wetlands that use macrophytes embedded in floating rafts to treat water of ponds, lakes, and rivers. The primary treatment mechanisms are provided by the roots, rhizomes, and attached biofilms, which are in direct and continuous contact with water^13^.

Plants release oxygen during daylight via their roots and rhizomes, influencing the redox potential in the water column^14^. This affects nitrogen transformation, oxidation of phytotoxins, and aerobic degradation of organic matter by microorganisms. Furthermore, root exudates impact biological processes such as denitrification^15^.

FTWs have been proven to remove nutrients and metals from water. Di Luca et al.^16^ demonstrated the feasibility of *Typha domingensis* in removing total phosphorus, ammonium, and nitrate. Mohsin et al.^17^ reported that *Iris pseudacorus* and *Phragmites australis* are applicable to nutrient and Cd removal. Ladislas et al.^18^ reported high Zn and Ni translocation in *Juncus effuses* and *Carex riparia* while Chen et al.^19^ proved Cr (VI) biosorption by *Typha angustifolia.* Di Luca et al.^4^ and Maine et al.^20,21^ revealed that *Pistia stratiotes*, *Salvinia herzogii*, and *Typha domingensis* had a high capacity for Cr(III) removal in an acidic environment. The same authors observed that Salvinia herzogii has the largest removal effect for Cr(VI) in the same acidic pH, up to 28%, although the tests revealed a detrimental influence on growth and general senescence. On the other hand, it has been widely reported that Cr is toxic to plants and may harm biomass development and tissue growth, raising concerns about the feasibility of phyto-techniques in Cr remediation^22,23^.

Several research gaps remain in the use of FTWs for water treatment, including species selection, sizing, system hydraulics, contaminant loading rate, management strategy, wildlife pressure, plan-microbiome interactions, and the rate of uptake and release of essential (nutrients and macro elements) and non-essential elements (e.g., heavy metals) over time^24^. Additionally, it is important to understand how Cr(III) is captured by wetland plants under natural pH conditions. In neutral and slightly alkaline environments (pH 7–8), Cr forms insoluble hydroxides that precipitate rapidly. The hypothesis of this study is that in FTWs, the precipitates formed on the underwater parts of plants can be later taken up by the plants. Therefore the aim of this study are to (1) compare the effect of different cosmopolitan species of perennials (common reed—*Phragmites australis,* Pa and yellow flag iris—*Iris pseudacorus,* Ip) on the performance of FTWs in removing Cr(III) and nutrients, (2) assess the bioaccumulation of Cr(III) in the different plant parts, (3) determine what portion of precipitated Cr(III) is actually taken up by plants in the overall mass balance under controlled conditions, and (4) verify the plants’ anatomical response on a tissue level to concentrations of Cr(III) in water within the range of wastewater discharge limits. The findings of this study may contribute to the advancement of FTWs as a sustainable technology for the final purification of industrial wastewater streams.

## Materials and methods

### Experimental setup

**Figure 1:**
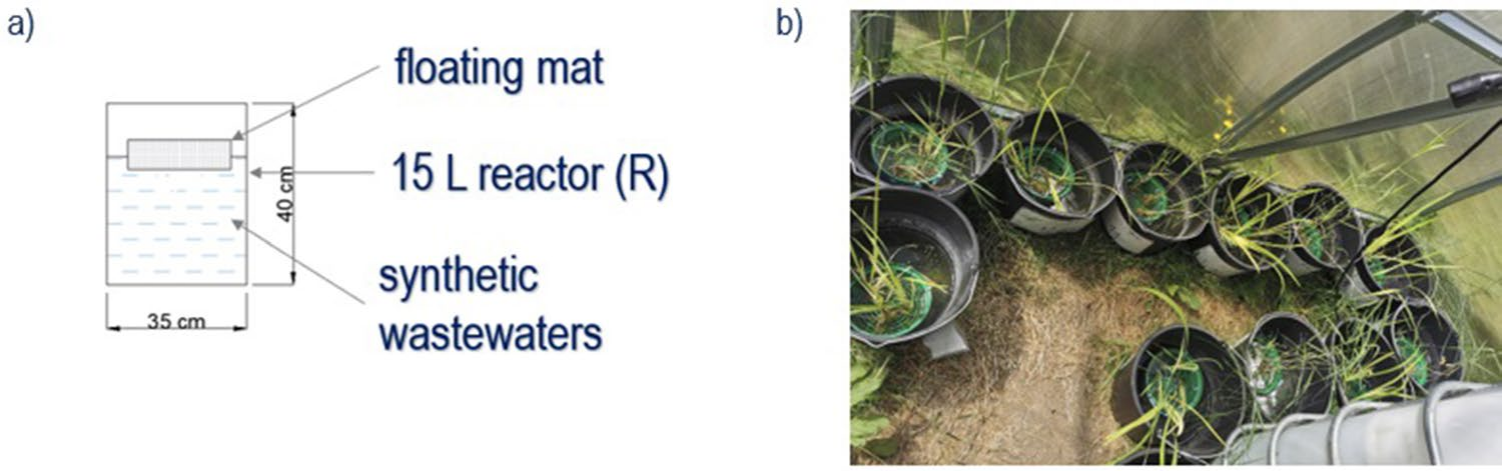
(**a**) A single reactor design with a floating treatment wetland and (**b**) the vegetative phase during the main experiment trial in the greenhouse.

*Iris pseudacorus* (Ip; yellow flag iris) and *Phragmites australis* (Pa; common reed) seedlings were purchased from a plant nursery and maintained as stock cultures in greenhouse pools. Plants have been marked with EU plant passport as documented in Table S.1. Both species were selected due to prolific growth potential. Before transplanting, cuttings were properly cleansed with water. Three cuttings of Ip and Pa were planted on artificial floating islands in each reactor (four with Ip, four with Pa) that were fed with tap water refilled every 7 days throughout the two-month acclimation phase (April and May 2021). A scheme of single reactor with FTW is shown in Fig. 1a. The plants were healthy developed on the day the experiment began; cuttings dimensions were as follows: 48–68 cm steams and 5–10 cm roots of *Phragmites australis* and 35–47 cm leaves and 5–7 cm roots of *Iris pseudacorus*.

Main experiment: The experimental reactors containing either *Phragmites australis* (Pa) or *Iris pseudacorus* (Ip) were fed with 10 L of synthetic wastewater characterized in Table S.1. To the control reactors, referred to as Ip-contr and Pa-contr for species Ip and Pa, respectively, only nitrogen and phosphorus were added. In three different treatments for each species, 500 µg/L, 1000 µg/L, and 2000 µg/L Cr was added as Cr(NO_3_)_3_·9H_2_O (nr cas 7789–02-8, ICP-MS-13N-R-5, Merck). These treatments were labeled as Ip-Cr500, Pa-Cr500, Ip-Cr1000, Pa-Cr1000, Ip-Cr2000, and Pa-Cr2000. The Cr concentrations were selected based on Cr levels observed in Polish lakes and rivers, which were markedly higher than the Cr discharge limits for wastewater^6^. The experiment lasted 50 days, from June 2nd to July 21st, 2021. The solution was sampled at the start of the experiment and after 10, 20, 35, and 50 days.

Plant samples of Ip and Pa from Ip-Cr500, Pa-Cr500, Ip-Cr2000, Pa-Cr2000 were harvested at the beginning (random selection of 5 seedlings as representative collected at the end of acclimation phase) and at the end of the experiment (all produced biomass) for Cr concentration studies. Collected biomass was thoroughly washed before further processing. In this study Cr was added as Cr(III) to the solutions, while further determined as total Cr in liquids and plant material. The pH of the solutions was not altered to maintain conditions similar to those found in natural aquatic ecosystems. The initial pH ranged between 7.34 and 8.03. The experiment was conducted under natural light and temperature in Gdansk, Poland under a transparent plexiglass cover to shield from rainwater (Fig. 1b). The average temperature during the experimental period was 20 °C.

Supporting experiment: Similar experiment as described as “Main experiment” was performed in the semi-controlled laboratory conditions with a constant air temperature of 20 ± 2 °C and artificial lightening (LED grow 10 W) with photo period 12/12 h. The experiment lasted 10 days, from September 19th to September 29th, 2023. Three different treatments of Cr for each species, 500 µg/L, 1000 µg/L, and 2000 µg/L Cr were prepared and repeated in duplicate runs. Control without plants has been established for all treatments. The initial pH ranges from 7.1 to 7.6. A period of the first 10 days was analyzed; the sampling of liquids was performed in intervals 0 min, 30 min, 1 h, 2 h, 8 h, 24 h, and 10 day.

### Chemical analyses

All protocols were carried out in accordance with the relevant guidelines and regulations, which are detailed in Table S.1.

Nutrient concentrations (as total nitrogen and orthophosphate) in liquid samples were analyzed using cuvette tests with a spectrophotometer (DR3900, Hach Lange). Plant material was digested with a mixture of HNO_3_, HCl, and H_2_O_2_ (all reagents for ultra-trace analysis). Chromium concentration in water and plant digests was determined using the ICP-OES method and an Agilent 5800 VDV spectrometer.

### Performance of floating treatment wetlands

Removal efficiency ($RE, \%$) of TN, PO_4_-P, and Cr were calculated as (1)^24^:

1 $$RE = \frac{{C_{0} - C_{t} }}{{C_{0} }} \cdot 100\left[ {\text{\% }} \right],$$

where $C_{0}$ and $C_{t}$ are initial and final concentrations of the element in medium (µg/L).

The removal rate ($RR$, µg/m^2^/day) of the floating treatment wetland area was calculated as (2)^24^:

2 $$RR=\frac{\left({C}_{0}-{C}_{t}\right)\cdot V}{{A}_{f}\cdot t}\left[\frac{\mu {\text{g}}}{{{\text{m}}}^{2}\cdot {\text{day}}}\right],$$

where $V$ is a volume of water body (m^3^; 0.01 m^3^), ${A}_{f}$ is a surface area of the floating treatment wetland (m^2^; 0.05 m^2^), and $t$ is a reaction time (day).

### Chromium impact on plants

#### Growth and biomass inhibition rates

Plant growth rates were calculated as^25^:

3 $${\mu }_{0-t}=\frac{ln\left({N}_{t}\right)-ln\left({N}_{0}\right)}{{\Delta t}_{0-t}}\left[\frac{{\text{leaves}}}{{\text{day}}}\right]$$

where ${\mu }_{0-t}$ is an average specific growth rate from initial “0” to final time “t”, ${N}_{t}$ is a number of leaves observed in the test or control vessel at time “t”, ${N}_{0}$ is a number of leaves observed in the test or control vessel at time “0”, and ${\Delta t}_{0-t}$ is the time from start to the end of the testing period.

The dry biomass, stem length, and root length tolerance indices ($DBTI$, $SLTI$, and $RLTI$, respectively) were calculated according to formulas (4–6)^16,26,27^:

4 $$DBTI=\frac{dry \;biomass \;of \;treated \;plant \left[g\right]}{dry \;biomass \;of \;control \;plant \left[g\right]}\cdot \;100 \left[{\%}\right]$$

5 $$SLTI=\frac{stem \;length \;of \;treated \;plant \left[cm\right]}{stem \;length \;of \;control \;plant \left[cm\right]}\cdot \;100 \left[{\%}\right]$$

6 $$RLTI=\frac{root \;length \;of \;treated \;plant \left[cm\right]}{root \;length \;of \;control \;plant \left[cm\right]}\cdot \;100 \left[{\%}\right]$$

#### Accumulation and transport of Cr in the plant

Bioconcentration factor (BCF) and translocation factor (TF) were used to evaluate the uptake of Cr by plants. Both factors are widely used in verifying plant species tolerance to high heavy metals in a soil substrate^28^. BCF may be easily applied to hydroponics. The roots/solution BCF was determined using Eq. (7)^17^:

7 $$BCF=\frac{{C}_{roots}}{{C}_{solution}}$$

where ${C}_{roots}$ is the concentration of metal in roots (µg/g d.w.) and ${C}_{solution}$ is the concentration of metal in solution (µg/L). BCF is a simple and reliable method for determining the relative bioavailability of heavy metals to plants^29^.

The plant efficiency in translocating accumulated metal from roots to shoots was calculated as Translocation Factor (TF)^28^:

8 $$TF=\frac{{C}_{AWP}}{{C}_{BWP}}$$

where ${C}_{AWP}$ and ${C}_{BWP}$ are the concentration (µg/g d.w.) of metal in above water parts of plants (AWP) and below water parts of plants (BWP), respectively. A TF value greater than one indicates that metal translocation from BWP to AWP is efficient^17^.

#### Light microscopy

Cross-sections of roots were examined using a light microscope (Biolar, PZO Poland) at 5 × and 10 × magnification of lens and 10 × magnification of eyepiece. Several (at least 3) roots fragments about 1 mm in size were taken from separate cuttings in each treatment; based on several micrographs from each treatment, consistent results and conclusions are presented. Free-hand sectioning method was used to prepare semi fine sections of plants that were stained with a 1% Toluidine Blue solution before examination.

### Data processing and statistical analysis

Plant seedlings in the floating treatment wetlands were randomly selected. Raw and processed data were collected in the dataset (10.34808/qvza-7781) and spreadsheet with Microsoft Excel to organize and summarize the data. A minimum, maximum, and average value for nutrients and Cr concentration in liquids were determined. The analysis of variance (ANOVA) was run using SPSS (ver. 27, NY, USA) to examine the effects of Cr treatments on studied plant species (height, biomass production, root length, no. of leaves) and Cr accumulation at a significance level *p* < 0.05 followed by the Tukey honestly Significant Difference (HSD) test. The results are presented as the means ± standard error (SE) of three replicates. The concentrations of Cr in plant materials were measured in homogenized and mixed samples of harvested biomass from duplicates.

## Results and discussion

### Removal of nitrogen and orthophosphate

**Figure 2:**
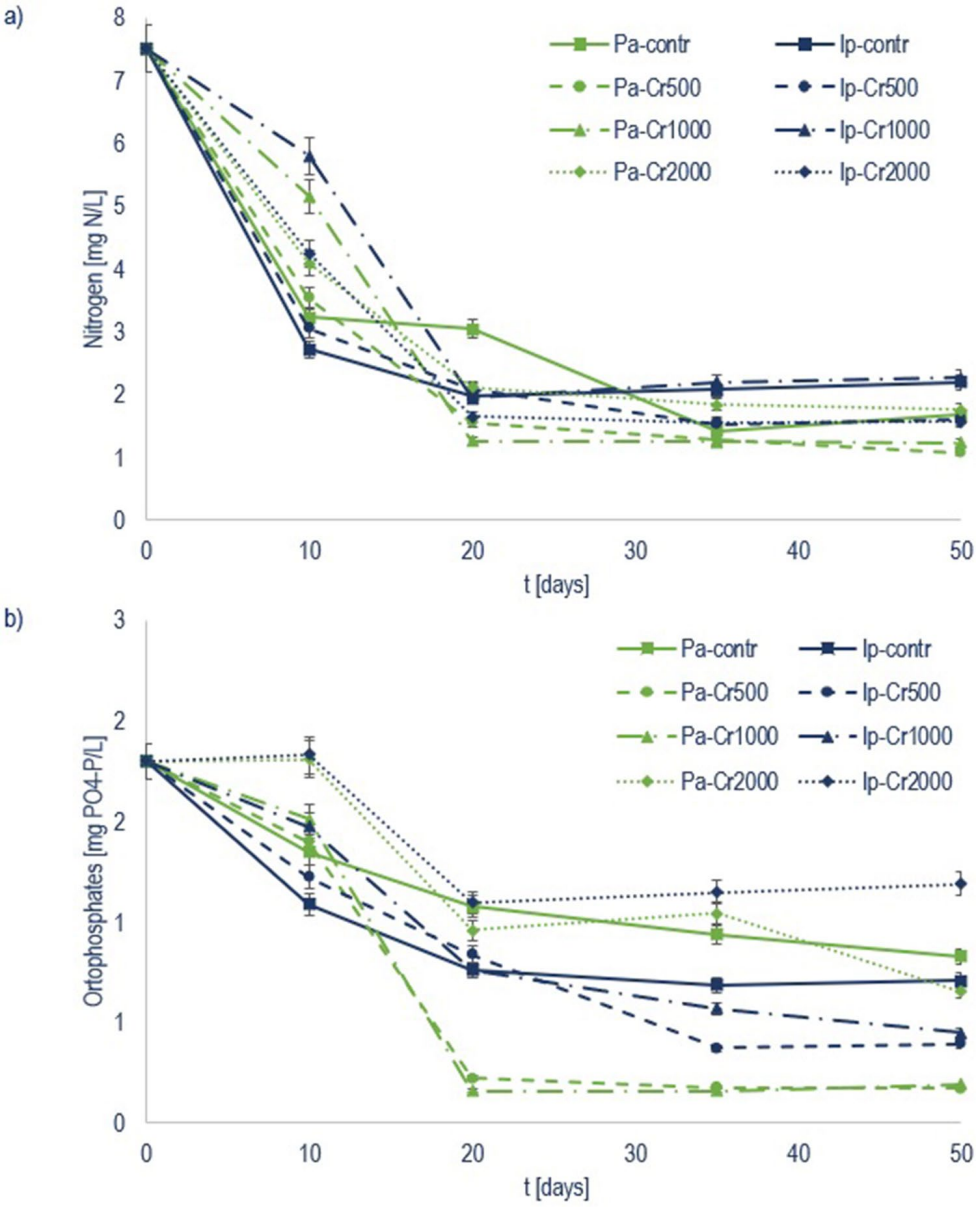
Nutrient concentrations (in mg/L) of (**a**) nitrogen (TN) and (**b**) orthophosphate (PO_4_-P) during 50 days of trial in control, Cr500, Cr1000, and Cr2000 treatments with *Phragmites australis* (Pa) and *Iris pseudacorus* (Ip) embedded in floating treatment wetlands.

Removal of nitrogen (TN) and orthophosphate (PO_4_-P) by floating treatment wetlands (FTWs) planted with *Phragmites australis* (Pa) and *Iris pseudacorus* (Ip) is shown in Fig. 2a,b. At the end of the experiment, 78% and 71% of TN were removed from the control treatments for Pa and Ip, respectively. In the Cr treatments, 70–86% of TN was removed (Table S.2). The highest TN removal occurred during the first 20 days, with the highest removal rate in the first 10 days 0.09 mg/m^2^/day for Pa and 0.10 mg/m^2^/day for Ip in controls. In the Cr treatments, the TN removal rate decreased in the following order (mg/m^2^/day): Cr500 (0.08-Pa; 0.09-Ip) > Cr2000 (0.07 Pa and Ip) > Cr1000 (0.05-Pa; 0.03-Ip). After 20 days, the removal rate dropped from 0.06 to 0.04 mg/m^2^/day, likely due to a deficiency of bioavailable nitrogen. In the controls, 54–60% of PO_4_-P was removed (Table S.3). The highest PO_4_-P removal was observed in the Cr500 and Cr1000 treatments (90% and 89% for Pa, and 78% and 75% for Ip). In Cr2000 with Pa, a higher PO_4_-P removal (64%) was observed compared to the control. Throughout the study period, the removal rate maintained at about 0.01 mg/m^2^/day in all treatments. In two subsequent trials in FTWs planted with five plant species (*Agrostis alba*, *Canna* × *generalis, Carex stricta*, *Iris ensata*, and *Panicum virgatum)*, Spangler et al.^30^ reported TN removal of 38–82% after the first trial and 13–60% after the second, while phosphorus removal was between 26–65% and 27–63%. Removal of TN varies greatly between plant species, from 8% for *Paphiopedilum barbatum*^31^ to 91% for *Oenanthe javanica*^32^. Reported removal of total phosphorus ranged between 4% for *Juncus effusus*^33^ and 92% for *Iris pseudacorus*^34^.

### Cr (III) removal in water

Given the negligible solubility of Cr(III) at neutral and slightly alkaline pH (7–8.5), the most likely removal process of dissolved Cr in liquid phase was precipitation on the surfaces of the reactors, as well as on plant roots hanging in the water. The removal efficiency of initially dissolved Cr reached up to 97–99% after 1 h (Table 1). According to Medina et al.^35^ and Tadesse et al.^36^, Cr(III) is quickly precipitated as Cr(OH)_3_ at pH 6–9, with optimum between 8.0 and 8.5. The supporting experiment showed that the removal efficiency of Cr exceeded 50% after 30 min in all Cr treatments, with and without plants. In the 50-day main experiment, there were no differences in dissolved Cr concentrations between treatments.

**Table 1:** Chromium concentration (mg/L) and removal efficiency (%) in floating treatment wetland reactors planted with Phragmites australis (Pa) and Iris pseudacorus (Ip) at different initial Cr concentrations for 50 days (“main experiment”) and 10 days (“supporting experiment”) (means and averages of 3 replicates).

Main experiment														
No.	Metal concentration [µg/L]										Removal efficiency [%]			
	0 min	± SD	10 days	± SD	20 days	± SD	35 days	± SD	50 days	± SD	10 days	20 days	35 days	50 days
Pa-Cr500	500	100	6.1	1.0	8.2	2.0	14.3	3.0	15.3	3.0	99	98	97	97
Ip-Cr500	500	100	10.0	2.0	10.0	2.0	12.0	2.0	13.0	3.0	98	98	98	97
Pa-Cr1000	1000	180	6.6	1.0	9.9	2.0	18.7	3.0	20.9	4.0	99	99	98	98
Ip-Cr1000	1000	190	8.3	2.0	11.5	2.0	17.7	4.0	32.3	6.0	99	99	98	97
Pa-Cr2000	2000	360	7.8	1.0	10.0	2.0	17.8	3.0	21.1	4.0	99	99	99	99
Ip-Cr2000	2000	370	9.6	2.0	11.8	2.0	13.9	3.0	18.2	3.0	99	99	99	99

Supporting experiment														
No.	Metal concentration [µg/L]												Removal efficiency [%]	
	0 min	± SD	30 min	± SD	1 h	± SD	2 h	± SD	24 h	± SD	10 days	± SD	30 min	1 h
Pa-Cr500	500	100	202	45	15	3.0	15	3.0	12	3.0	10	2.0	60	97
Ip-Cr500	500	100	197	38	18	2.0	18	2.0	15	2.0	15	2.5	61	96
Pa-Cr1000	1000	190	501	95	20	3.5	20	3.0	14	3.0	12	2.5	50	98
Ip-Cr1000	1000	170	480	99	15	3.0	15	3.0	18	3.0	18	3.0	52	99
Pa-Cr2000	2000	310	998	350	18	4.0	18	2.0	15	2.0	21	3.0	50	99
Ip-Cr2000	2000	330	1054	410	22	3.0	22	4.0	18	3.0	28	3.0	47	99
Cr500_cont	500	80	252	34	12	3.0	10	2.0	15	2.0	15	2.0	50	98
Cr1000_cont	1000	150	542	112	18	2.0	14	2.0	15	3.0	18	2.0	46	98
Cr2000_cont	2000	350	1132	178	32	3.0	33	3.0	28	5.0	31	6.0	43	98

### Role of macrophytes Cr(III) removal

The initial Cr concentrations in Pa and Ip were very low; 0.88 ± 0.22 and 0.62 ± 0.16 mg/kg d.w. in above water parts (AWP); 1.22 ± 0.30 and 0.62 ± 0.15 mg/kg d.w. in below water parts (BWP), respectively (Table 2). At the end of main experiment, both plant species exhibited high Cr contents in the Cr500 and Cr2000 treatments, with the highest values observed for AWP and BWP for Ip (Cr1000 was not examined). In both species, concentration in the BWP was markedly higher than in the AWP. This observation is in line with research developed by Maine et al.^20^ for Pistia stratiotes and Salvinia herzogii for Cr(III). The patterns of Cr accumulation (i.e., concentration × biomass) differed between plant parts (Table 2 and Fig. S.2). Despite having nearly twice lower biomass development, Ip accumulated more Cr in BWP than Pa in the Cr500 treatment. Conversely, Pa exhibited a higher Cr accumulation in BWP in the Cr2000 treatment, likely due to Ip weaker biomass development. Both Pa and Ip showed increased Cr accumulation in BWP compared to the initial Cr accumulation, with 70 and 209 fold increases in the Cr500 treatment and 267- and 413 fold increase in Cr2000 treatment for Pa and Ip, respectively. Chromium accumulation for Pa in the AWP did not increase in the Cr500 treatment compared to the control, while showed an eightfold increase. Compared to the control, Cr accumulation in the AWP increased by 4 times for Pa and 14 times for Ip.

**Table 2:** Chromium concentration and accumulation in *Phragmites australis* (Pa) and *Iris pseudacorus* (Ip) at the end of the experiment, as well as bioconcentration factor (BCF) and translocation factor (TF) for control, Cr500, and Cr2000 treatments.

Treatment	Cr in plants [mg/kg d.w.]		Cr in plants [µg]		BCF	TF
	Concentration	± SD	Accumulation	± SD		
AWP						
Pa-contr	0.88^a^	0.22	4.5^a^	1.1		–
Pa-Cr500	1.04^a^	0.26	3.8^a^	0.9		0.01
Pa-Cr2000	5.12^b^	1.28	19^b^	4.7		0.01
Ip-contr	0.62^a^	0.16	3.9^a^	1.0		–
Ip-Cr500	7.82^b^	1.96	29^b^	7.2		0.03
IpCr2000	25.2^c^	6.3	53^c^	13.3		0.05
BWP						
Pa-contr	1.22^a^	0.30	11.0^a^	2.7	–	
Pa-Cr500	108^b^	27	766^b^	191	1.53	
Pa-Cr2000	364^c^	91	2297^c^	574	1.15	
Ip-contr	0.62^a^	0.15	3.2^a^	0.8	–	
Ip-Cr500	280^b^	70	851^b^	213	1.70	
IpCr2000	537^c^	134	1321^b^	330	0.66	

Superscript letters indicate significant differences among Cr treatments for each species at *p* < 0.05.

There are different explanations for the higher Cr accumulation in BWP; firstly, according to Zayed et al.^37^, Cr translocation from roots to shoots in grass family (*Poaceae*) species is limited, resulting in Cr storage in roots. Secondly, the higher Cr accumulation in roots than in shoots and leaves may be due to the immobilization processes of Cr in root cortex cells^38^. Finally, such behavior may be due to the natural toxicity response of plants^22^. Cr(III) plays no essential role in plant metabolism, and is taken up passively^1^. The poor translocation from BWP to AWP is reflected in low values of TF. Transfer of Cr from plant roots to aerial tissues is usually limited, though it is highly dependent on the chemical form of Cr within plant tissues^1^; negligible translocation was reported for Cr(III), while higher translocation to aerial parts for Cr(VI)^20^ probably due to detrimental and damaging features of Cr(VI). As a result, Cr(III) concentrations in roots are typically 100 times higher than in aerial parts. Caldelas et al.^39^ found the highest Cr(III) concentrations in cell walls of the cortex (C) in roots as well as in cytoplasm and intercellular spaces of rhizomes in *Iris pseudacorus* species. Liu et al.^40^ found that majority of Cr(III) (83%) was sorbed on cell walls in the roots, whereas 58% of Cr(III) was sequestered in the vacuoles and cytoplasm of the leaves. The formation of insoluble Cr compounds within plants may explain why more Cr is sequestered in the roots than in the leaves. Singh et al.^41^ suggested that Cr sequestration in the vacuoles of root cells is a defence mechanism in plants. Both studied species took up high amounts of Cr despite a decrease in growth rate and biomass, suggesting they may be suitable for use in floating treatment wetlands for Cr(III) removal.

**Figure 3:**
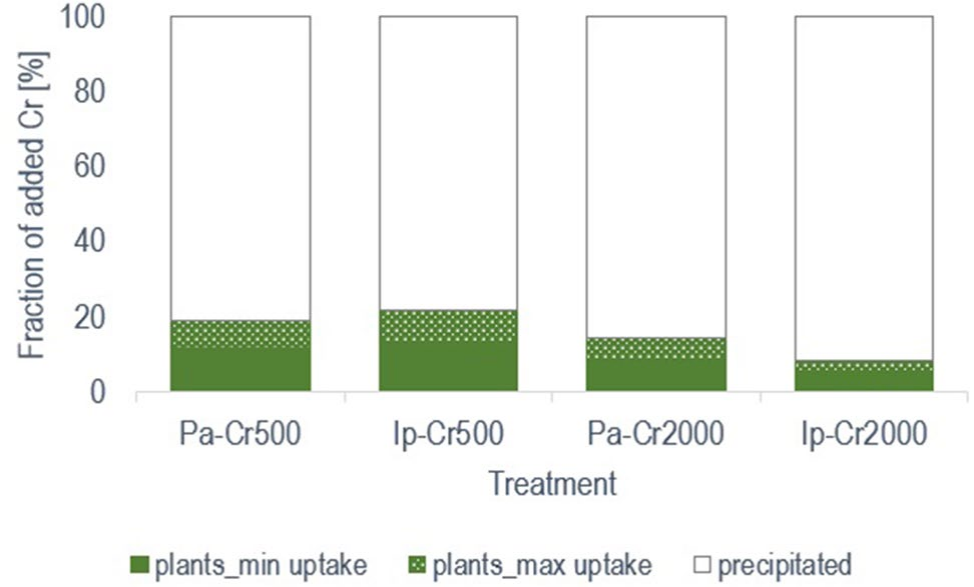
Percentage of total added Cr(III) bound by *Phragmites australis* (Pa) and *Iris pseudacorus* (Ip) in a floating treatment wetland, as determined by a mass balance. ‘plants’ refers to Cr bound in plant biomass, while ‘precipitated' means Cr precipitated or sorbed on the reactor walls.

Dissolved Cr(III) quickly precipitated as insoluble hydroxides that were subsequently partly uptaken by plants in FTW microcosms. In reference to mass balance of each Cr treatments, after 50 days of exposition Pa accumulated max 19% in the Cr500 treatment, and 14.5% in the Cr2000 treatment. For Ip, these numbers are 22 and 9% (Fig. 3). Moreover, the obtained results refer to the actual Cr content in the tissues, because at the end of the experiment the collected biomass was thoroughly washed which removed Cr precipitated on roots that were no bound in cell walls. Cr uptake by plants are mediated by root exudates and microorganisms^42,43^. Probably, the mass of Cr bound by plants would be more beneficial if plant roots and rhizomes development was better or if the coverage ratio was higher. In this study, the ratio of below water part dry matter [g] to solution volume [L] was in the ranges 0.6–0.9 g/L for Pa and 0.25–0.5 g/L for Ip.

### Plants growth, biomass development, and anatomical examination

The dry biomass production of both plant species was similar under different Cr doses (Table 3). Chromium treatment caused a decrease in dry biomass production, consistent with the literature^27,44^. Shoot growth (above water parts—AWP) was reduced in all Cr treatments for both species when compared to the control, except in Pa, where shoot development was 10% better in Cr2000 than in the control. UdDin et al.^22^ also reported different growth response to Cr(III) treatment in *Solanum nigrum* and *Parthenium hysterophorus,* observing that low levels of Cr(III) inhibited shoot growth in *Parthenium hysterophorus*, while high levels of Cr(III) promoted shoot biomass development in both *Solanum nigrum* and *Parthenium hysterophorus*. This could be due to the secretion of root exudates at higher Cr(III) levels, which Javed et al.^45^ suggest are are responsible for metal detoxification in plants through modulation of pH in the rhizosphere. Higher Cr doses may also have a differential effect on the microbiome, favoring the survival and growth of tolerant plant growth-promoting rhizobacteria, which could lead to the development of specific rhizosphere symbiosis.

**Table 3:** Biomass production, plant height, and number of leaves of *Phragmites australis* (Pa) and *Iris pseudacorus* (Ip) at the end of the experiment, as well as growth and biomass inhibition rates in the control, Cr500, Cr1000, and Cr2000 treatments.

Characteristic	Unit	*P. australis*	*I. pseudacorus*
Biomass			
Dry AWP treatment			
Control	[g]	5.14 ± 0.2^a^	6.22 ± 0.12^a^
Cr500	[g]	3.65 ± 0.23^b^	3.69 ± 0.56^b^
Cr1000	[g]	1.82 ± 0.16^c^	1.16 ± 0.18^c^
Cr2000	[g]	3.64 ± 0.52^b^	2.11 ± 0.06^d^
Dry BWP treatment			
Control	[g]	8.99 ± 0.39^a^	5.15 ± 0.14^a^
Cr500	[g]	7.09 ± 0.13^b^	3.04 ± 0.39^b^
Cr1000	[g]	2.12 ± 0.33^c^	1.32 ± 0.08^c^
Cr2000	[g]	6.31 ± 0.35^d^	2.46 ± 0.25^b^
Plant height			
AWP (start/end)			
Control	[cm]	48/68	35/44
Cr500	[cm]	66/88	47/52
Cr1000	[cm]	51/57	45/54
Cr2000	[cm]	57/94	42/46
BWP (start/end)			
Control	[cm]	7/13	7/12
Cr500	[cm]	7/26	6/13
Cr1000	[cm]	5/12	5/15
Cr2000	[cm]	7/19	10/14
No of leaves			
Control		9 ± 2^a^	7 ± 2^ab^
Treatment Cr500		18 ± 2^b^	10 ± 2^a^
Treatment Cr1000		20 ± 2^b^	5 ± 2^b^
Treatment Cr2000		16 ± 4^b^	8 ± 4^ab^
Growth rate ${\upmu }_{0-{\text{t}}}$			
Control	[leaves/day]	0.01	0.02
Treatment Cr500	[leaves/day]	0.03	0.02
Treatment Cr1000	[leaves/day]	0.03	0.01
Treatment Cr2000	[leaves/day]	0.02	0.02
DBTI			
Treatment Cr500	[%]	76	59
Treatment Cr1000	[%]	28	22
Treatment Cr2000	[%]	70	40
SLTI			
Treatment Cr500	[%]	129	76
Treatment Cr1000	[%]	84	123
Treatment Cr2000	[%]	138	105
RLTI			
Treatment Cr500	[%]	200	108
Treatment Cr1000	[%]	92	125
Treatment Cr2000	[%]	146	117

Different letters indicate significant differences (*p* < 0.05) at 95% confidence interval (n = 3) among Cr treatments.

*AWP* means in this case the height of the stem [cm]; while *BWP* means in this case the height of roots [cm], *DBTI* dry biomass tolerance index [%], *SLTI* stem length tolerance index [%], *RLTI* root length tolerance index [%].

At the end of the experiment, Pa had approximately twice as many leaves as Ip, but the leaves were smaller in all Cr treatments compared to the control. Growth rate measurements (in terms of leaves produced per day) also indicated that Pa is more tolerant to high Cr(III) doses. The growth rate for Pa (leaves/day) decreased in the following order: Cr500 = Cr1000 > Cr2000 > control. The number of leaves and growth rate parameters for Ip were similar to those of Pa. Üçüncü et al.^25^ reported high growth rate parameters for *Lemna minor* in a mixture of Cr(III) + Pb(II) + Cu(II) (10.4/0.2/3 [mg/L])–0.06 leaves/day and Cr(III) + Pb(II) (20.8/0.2 [mg/L])–0.10 leaves/day.

Stress tolerance indices (TI) with respect to total dry biomass (DBTI), stem length (SLTI), and root length (RLTI) were used to assess the stress tolerance potential of plants under Cr stress. The DTPI after 50 days of exposure to different Cr(III) concentrations was lower than in the control treatment, indicating diminished stress tolerance. However, the STLI and RTLI were lower than the control only in the Cr1000 treatment for Pa, and greater than 100% in other cases. Mohsin et al.^16^ reported that the DBTI of Ip was not inhibited by different cadmium treatments (1000, 2000, and 4000 mg/L) and that the DBTI of Pa was not inhibited by the Cd 2000 mg/L treatment, indicating that both species have high tolerance towards Cd. UdDin et al.^22^ presented similar data, reporting that wild and wetland plants may play a role in hyperaccumulation of toxic metals and their conversion to non-toxic and immobile compounds under Cr(III) stress conditions.

**Figure 4:**
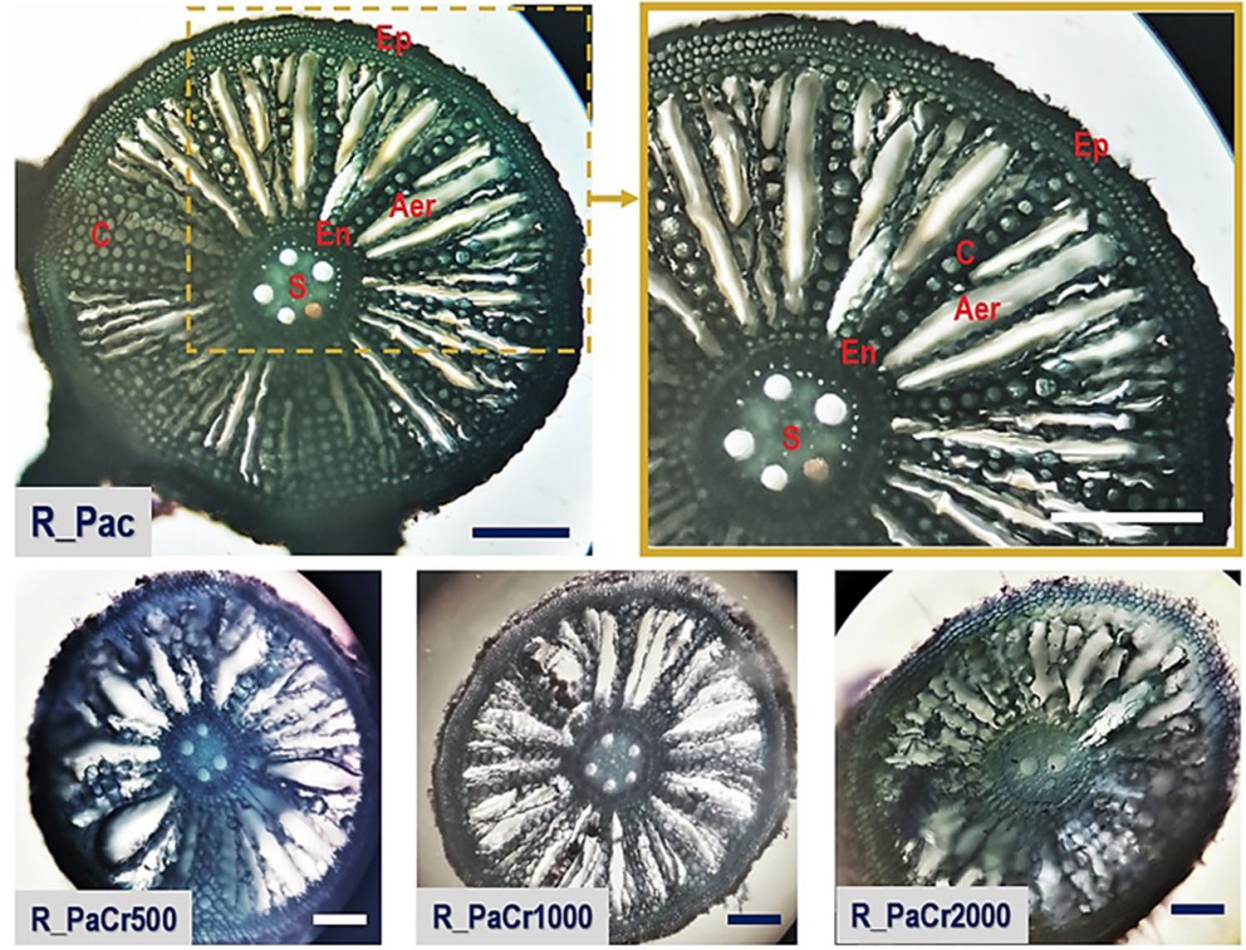
Light micrographs of the transverse roots section of *Phragmites australis* for control, Cr500, Cr1000, and Cr2000 treatments; The scale bar is 150 μm (mark: S—stele, En—endodermis, Aer—aerenchyma, C—cortex, Ep—epidermis).

A transverse section of the Pa control root (Fig. 4) reveals the external layer of the epidermis (Ep), the internal layer of the endodermis (En), the central cylinder (stele, S), cortex (C), and air spaces between them (aerenchyma, Aer). The root diameter is approximately 1 mm, which is consistent with the data reported by Baldantoni et al.^46^ (measured at 5–7 cm from the apex). In the Cr500 and Cr1000 treatments, no significant changes in the structure of the stele were revealed (quantitatively more vessel elements form the cylindrical S), while in Cr2000 vessel elements in stele arrangement is disrupted. In general, epidermal and endodermal cells are packed regularly without disturbances in all treatments. Cortex (C) is deformed in Cr treatments; air channels separated by several layers of parenchymal cells are larger than in the control. Also the decrease in the frequency of aerenchyma cells was observed as influenced by Cr. In general, an increase in aerenchyma space is frequently reported in Pa sediment roots as an adaptation feature to low oxygen availability in sediments, which promotes oxygen diffusion from the plant's aerial to underground tissues^47,48^.

**Figure 5:**
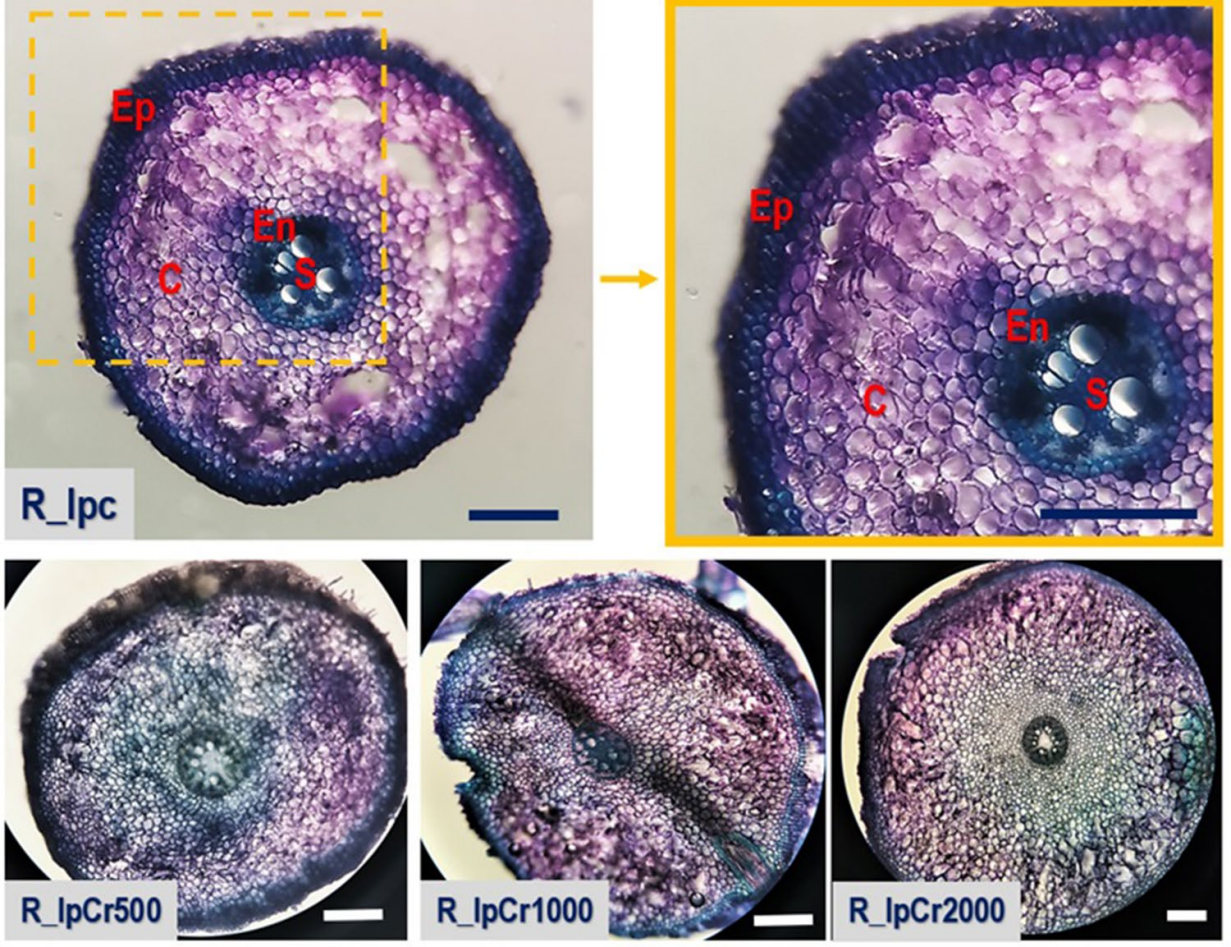
Light micrographs of the transverse roots section of *Iris pseudacorus* for control, Cr500, Cr1000, and Cr2000 treatments; The scale bar is 150 μm (mark: S—stele, En—endodermis, Aer—aerenchyma, C—cortex, Ep—epidermis).

Light micrographs of a transverse section through the root of Ip (Fig. 5) show a central vascular tissue (stele, S) surrounded by endodermis (En), cortex (C), and an outer piliferous layer bearing numerous water-absorbing root hairs (epidermis, Ep). Cortex (C) is multilayers; parenchyma is approximately 12 layered in control and even wider (18–22 layers) in Cr treatments. The increase in parenchyma layers was also reported by Mohsin et al.^16^ for Pa and Ip in Cd treatments. Parenchymal cells are large and oval-shaped. Water-conducting metaxylem vessels called trachea encircle a small, regular-patterned area of the parenchymatous pith's centre. Only in Cr2000 there are more xylem vessels with irregular arrangement in comparison to other treatments. In the Cr1000 treatment emerging lateral root formation is visible. To summarise, the light microscope revealed no histological changes in the roots of control or Cr500 and Cr1000 treated plants, with possible adaptation changes in stele observed in Cr2000.

## Conclusions

Contributions of *Phragmites australis* and *Iris pseudacorus* to Cr(III) removal in floating treatment wetlands was invetigated. Three treatments with different Cr(III) concentrations (500, 1000, and 2000 µg/L) were prepared. Within 1 h, the initial Cr(III) loading was almost completely (96–99%) removed from solution, mainly due to precipitation as hydroxides on the surfaces of plastic reactors and plant roots. Over 50 days, *Phragmites australis* and *Iris pseudacorus* were able to take up 9–19% and 5–22% of the added Cr, respectively. Chromium was mostly bound in below water parts of plants, with virtually zero translocation to aerial parts (translocation factor: 0.01–0.05). Decrease in dry biomass development and growth rate inhibition were observed for subsequent Cr(III) treatments in comparison to the control. Interestingly, Cr1000 caused stronger effects than Cr2000, possibly due to rhizosphere pH modulation with root secretion as a mechanism for metal detoxification at higher concentrations. Anatomical examination revealed no histological changes in the roots of the control, Cr500 and Cr1000 treatments. However, Cr2000 caused disruptions in the arrangement of vessel elements in the stele of *Iris* and changes in aerenchyma spaces formation in *Phragmites*.

Overall, both perennial emergent macrophytes are capable of accumulating Cr(III) without substantial damages to plants. This indicates that they could be suitable for Cr(III) removal from natural waters at neutral pH. Further research should focus on explaining the role of root exudates and rhizomicrobiome in Cr(III) binding and uptake by different plant species. Additionally, the effects of root and rhizome and plant exposure time on Cr(III) should be investigated.

### Supplementary Information

Supplementary Information.

## Data Availability

The dataset generated during the current study is available in the Bridge of Knowledge repository available under the link: Nawrot, N., & Wojciechowska, E. (2023). Chromium FTW dataset (1–) [dataset]. Gdańsk University of Technology. https://doi.org/10.34808/qvza-7781.
