# Supplementary material for: Chromium (III) removal by perennial emerging macrophytes in floating treatment wetlands
Source: Sci Rep. 2023 Dec 16;13:22417. doi: 10.1038/s41598-023-49952-y (PMC10725432; doi:10.1038/s41598-023-49952-y)
Supplement: Supplementary file 1 — Supplementary Information. [file 41598_2023_49952_MOESM1_ESM.pdf]

Supplementary material to the manuscript entitled:

Chromium (III) removal by perennial emerging macrophytes in floating treatment wetlands

Nicole Nawrot[1,*], Ewa Wojciechowska[1], Muhammad Mohsin[2], Suvi Kuittinen[2], Ari Pappinen[2], Karolina Matej-Łukowicz[1], Katarzyna Szczepańska[3], Agnieszka Cichowska[3], Muhammad Atif Irshad[4,5], Filip M.G. Tack[6]

[1] Gdansk University of Technology, Faculty of Civil and Environmental Engineering, Narutowicza 11/12, 80-233, Gdansk, Poland

[2] School of Forest Sciences, University of Eastern Finland, Yliopistokatu 7, P.O. Box 111, 80100 Joensuu, Finland

[3] Department of Environmental Protection, Gdynia Maritime University Maritime Institute, Gdynia, Poland

[4] Department of Environmental Sciences and Engineering, Government College University, Faisalabad 38000, Pakistan

[5] Department of Environmental Sciences, The University of Lahore, Lahore 54590, Pakistan

[6] Department of Green Chemistry and Technology, Ghent University, Faculty of Bioscience Engineering, B-9050 Ghent, Belgium

[*]Corresponding author: nicole.nawrot@pg.edu.pl

Table Supplement 1 Materials & methods supporting part

Specification	Description
Plant passports	A *Phragmites australis*<br>B PL-30/21/1582<br>C PW 3/GOR/11/2019/412403<br>D PL<br>A *Iris pseudacorus*<br>B PL-30/18/20385<br>C 3/20<br>D PL
Synthetic wastewaters	The initial ratio of total nitrogen to orthophosphate, TN:PO_4-P, was set at 4:1, corresponding to 7.5 mg/L `of TN and 1.8 mg/L of PO_4-P.<br>The stock solution of wastewaters was prepared as a mixture of ammonium nitrate NH_4NO_3, potassium dihydrogen phosphate KH_2PO_4, and in Cr-loaded reactors with chromium (III) nitrate $Cr(NO_3)_3 \cdot 9H_2O$.
Liquid samples analyses for TN, PO4-P, and Cr determination	TN and PO_4-P concentrations were analyzed with a spectrophotometer (DR3900, Hach Lange) using a cuvette test according to EN ISO 11905-1 Persulphate mineralization and EN ISO 6878 Phosphomolybdenum blue method. Liquid samples for Cr analyses were collected using a pure syringe, filtered with Millipore filters (0.45 μm), and stored in polypropylene tubes to which 100 μL of 65% HNO_3 (Suprapur, Merck) per 10 mL was added. The tubes were stored at 8 degrees Celsius. The Cr concentration in liquid samples was determined using ICP-OES (Agilent 5800 VDV, Agilent Technologies, Australia). An LGC6027 certified reference material (CRM, LGC Standards, Soft Drinking Water, LGC Standards) was analyzed to assess analytical accuracy. The average recovery rate was 100.1 %.
Plant material analyses for Cr determination	The samples were digested using nitric acid (65.5%, Suprapur, Merck), hydrochloric acid (36%, Suprapur, Merck), and hydrogen peroxide solution (30.9%, for ultra-trace analysis, Merck). Blanks and samples of certified reference material (CRM, LGC7162, Strawberry Leaves, LGC Standards) were prepared using the same digestion technique as the samples of Ip and Pa. Following digestion, the solutions were transferred quantitatively to 25 mL volumetric flasks and filled to full content with deionized water. Cr content in prepared samples was determined using the ICP–OES method and an Agilent 5800 VDV spectrometer. CRM determined the correctness of the analysis. Four separately generated CRM samples yielded findings that were compared to certified values. All of the data were within the manufacturer's uncertainty range. The accuracy was given as a percentage recovery, with mean value of 98.2 %.
Plants preparation for microscopic observation	Plant tissues were cut into semi-thin (0.5–1 mm) slices using a razor blade and placed in a watch glass filled with redistilled water (Milli-Q Ultrapure Water System, Merck). The cross-sections were soaked in water for 2-3 minutes before being immersed in the staining solution for one minute. Toluidine blue was used to distinguish between tissue structures (Parker et al., 1982). Sections were washed in redistilled water after staining, put on a clean glass slide with a drop of water and covered with a coverslip.

References:

Parker, A. J., Haskins, E. F., & Deyrup-Olsen, I. (1982). Toluidine Blue: A Simple, Effective Stain for Plant Tissues. *American Biology Teacher*, *44*(8), 487–489. https://doi.org/10.2307/4447575

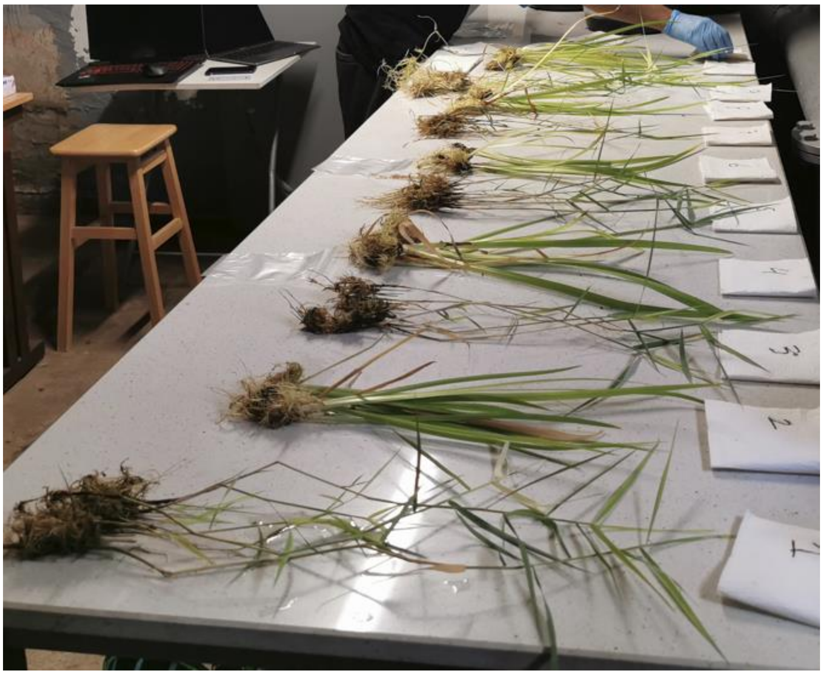

Figure Supplement 1 Plants harvested at the end of the main experiment

Table Supplement 2 Total nitrogen TN removal efficiency [%] and removal rate RR [mg/m^2/day] for control and Cr treatments

Reactor	TN removal efficiency - RE [%]				TN removal rate – RR [mg/m^2/day]			
	**10**	**20**	**35**	**50**	**10**	**20**	**35**	**50**
Pa-contr	57	59	81	78	0.09	0.05	0.04	0.02
Ip-contr	64	73	72	71	0.10	0.06	0.03	0.02
Pa-Cr500	53	79	83	86	0.08	0.06	0.04	0.03
Ip-Cr500	59	72	80	79	0.09	0.06	0.03	0.02
Pa-Cr1000	31	83	83	84	0.05	0.06	0.04	0.03
Ip-Cr1000	23	74	71	70	0.03	0.06	0.03	0.02
Pa-Cr2000	45	72	75	77	0.07	0.05	0.03	0.02
Ip-Cr2000	43	78	79	79	0.07	0.06	0.03	0.02

Table Supplement 3 Orthophosphate (PO_4-P) removal efficiency [%] and removal rate RR [mg/m^2/day] for control and Cr treatments

Reactor	TP removal efficiency [%]				TP removal rate – RR [mg/m^2/day]			
	10	20	35	50	10	20	35	50
Pa-contr	25	40	48	54	0.01	0.01	0.01	0.00
Ip-contr	40	58	62	60	0.01	0.01	0.01	0.00
Pa-Cr500	22	88	90	90	0.01	0.02	0.01	0.01
Ip-Cr500	32	53	79	78	0.01	0.01	0.01	0.01
Pa-Cr1000	16	91	91	89	0.01	0.02	0.01	0.01
Ip-Cr1000	18	58	68	75	0.01	0.01	0.01	0.01
Pa-Cr2000	-1	47	42	64	0.00	0.01	0.00	0.00
Ip-Cr2000	-2	39	36	34	0.00	0.01	0.00	0.00

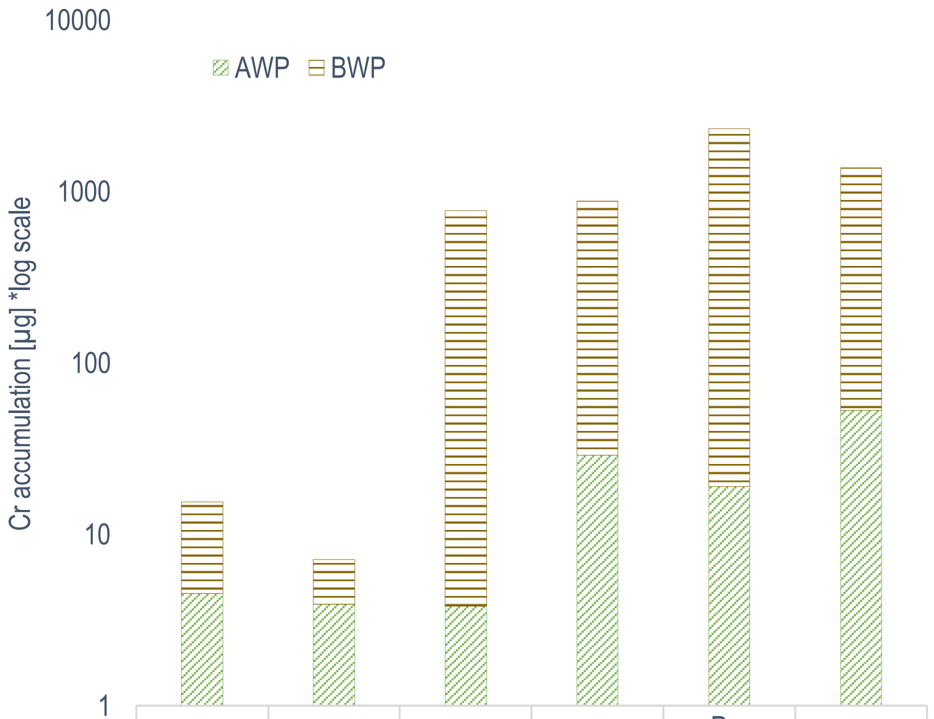

	Pa-cont	Ip-cont	Pa-Cr500	Ip-Cr500	Pa-Cr2000	Ip-Cr2000
BWP	11	3,2	766	851	2297	1321
AWP	4,5	3,9	3,8	29	19	53

Figure Supplement 2 Chromium accumulation [µg] in above water parts (AWP) and below water parts (BWP) of plants in different Cr treatments; Pa – *Phragmites australis*; Ip – *Iris pseudacorus*; *log scale
